# IDENTIFYING SALIENT TRAINING AND SUPPORT NEEDS WITHIN A STATEWIDE LIFELONG COMMUNITIES NETWORK

**DOI:** 10.1093/geroni/igz038.951

**Published:** 2019-11-08

**Authors:** Jennifer Crittenden, Patricia A Oh, Laura Lee

**Affiliations:** 1 UMaine Center on Aging, University of Maine, Bangor, Maine, United States; 2 University of Massachusetts, Boston, Boston, Massachusetts, United States; 3 Maine Community Foundation, Portland, Maine, United States

## Abstract

As the older adult population grows in the United States, the need for community planning approaches that respond to the needs of older adults is of increasing importance. As a result, lifelong community movements, encompassing models such as Age-Friendly Communities, Livable Communities, and “Aging-in-Place” initiatives are proliferating. Maine, the oldest state by median age, currently hosts the largest number of AARP designated Age-Friendly Communities efforts. Given the size of this network, the purpose of this study was to collect descriptive information about the status of existing lifelong communities initiatives, their training and support needs, and the desired format and configuration of future training programming. An electronic survey was distributed to community representatives from 76 lifelong communities initiatives throughout Maine. A total of 38 communities responded to the survey representing a response rate of 50%. The majority of respondents (80.4%) reported having a committee or other coordinating group guiding their work. A large portion have completed planning phase activities including hosting focus groups (79.5%), carrying out a survey (66.7%), and identifying a list of local assets (59%). Fewer have completed the plan drafting phase (17.9%). A majority reported receiving assistance from AARP (66%) and a regional educational consortium (66%). The areas with highest self-reported training needs (based on mean ratings) are: Volunteer recruitment and retention, specialized trainings on Age-Friendly Community topics, Dementia-Friendly Communities topics, and outreach and community engagement strategies. Implications will be discussed including optimal configuration of training and support for similar such networks.

